# Supplementary material for: Genetic architecture of individual meiotic crossover rate and distribution in Atlantic Salmon
Source: Sci Rep. 2023 Nov 22;13:20481. doi: 10.1038/s41598-023-47208-3 (PMC10665409; doi:10.1038/s41598-023-47208-3)
Supplement: Supplementary file 1 — Supplementary Information. [file 41598_2023_47208_MOESM1_ESM.pdf]

Supplementary

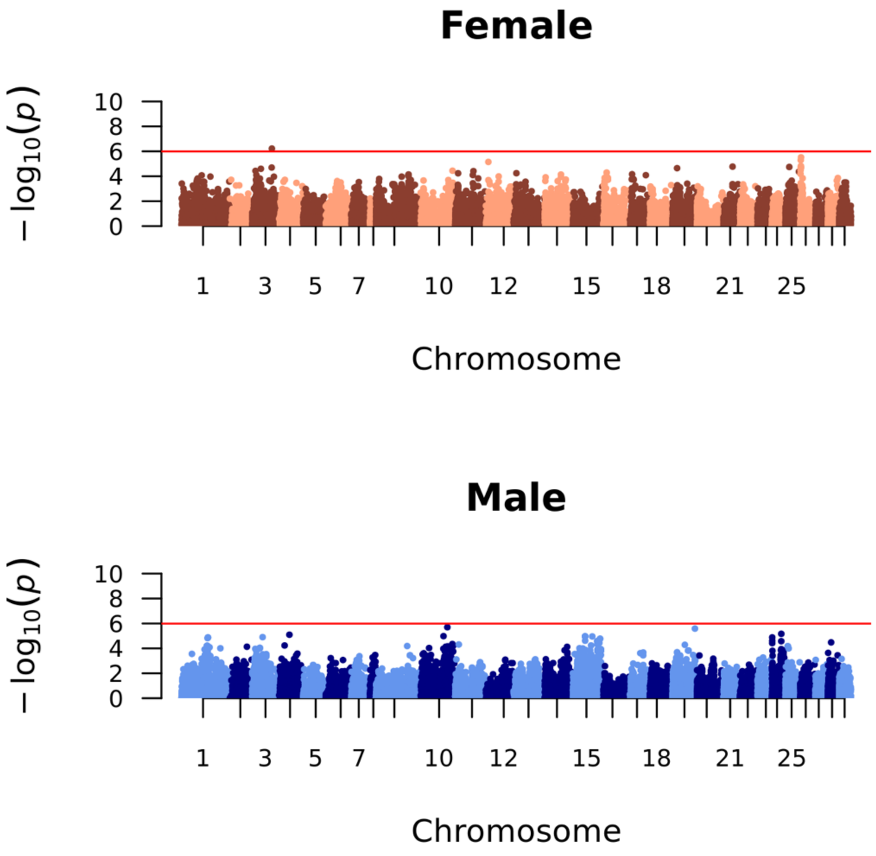

Fig S1. Manhattan plots of genome-wide association between markers and crossover count (CC). The red line is the genome-wide significance threshold using a Bonferroni correction. The Y axis is the negative logarithm of the p-values, and the x-axis is the physical positions of the markers with alternating colours for chromosomes 1-29 for males (A) and females (B).

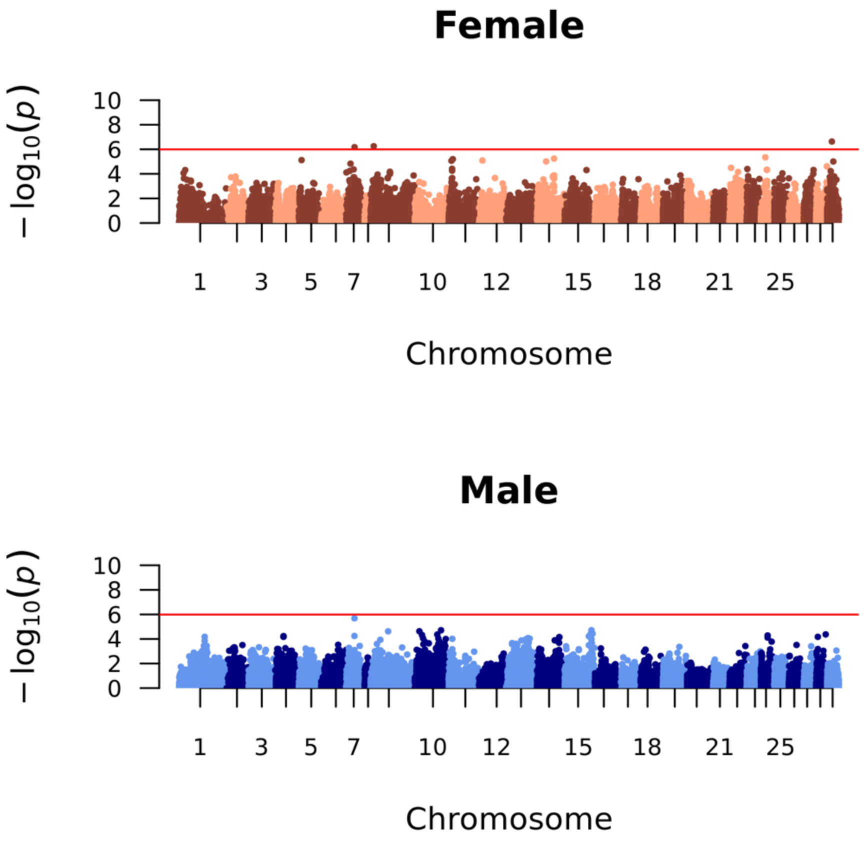

Fig S2. Manhattan plots of genome-wide association between markers and intrachromosomal shuffling ($\bar{r}$).
The red line is the genome-wide significance threshold = 0.05/Number of markers per analysis. The Y axis is the negative logarithm of the p-values, and the x-axis is the physical positions of the markers with alternating colours for chromosomes 1-29 for males (A) and females (B).

Table S1. Linkage map. Male and female genetic positions in cM for all SNP markers in the 35K dataset. See additional file "S1_Table.xls".

Table S2. Top 10 SNPs from GWAS on shuffling and crossover count in both sex

Trait	Sex	Chr	SNP name	bp	Freq	b	SE	p
CC	Male	10	Affx-87165681	97158781	0.216	0.936	0.197	2.00E-06
		19	Affx-986688163	80841039	0.209	-0.844	0.179	2.58E-06
		24	Affx-517031085	33571077	0.286	0.778	0.173	6.79E-06
		4	Affx-87621822	40639301	0.177	0.890	0.199	8.01E-06

		10	Affx-86913301	83219166	0.176	0.850	0.193	1.04E-05
		15	Affx-87001560	44530526	0.517	0.651	0.148	1.09E-05
		15	Affx-87481996	44534054	0.517	0.651	0.148	1.09E-05
		15	Affx-87658641	44541415	0.483	-0.651	0.148	1.09E-05
		15	Affx-98990447	44543717	0.483	-0.651	0.148	1.09E-05
		15	Affx-517023952	70320171	0.407	-0.656	0.149	1.10E-05
CC	Female	3	Affx-87260986	71736893	0.286	-0.643	0.129	6.00E-07
		26	Affx-517021201	3373590	0.327	-0.567	0.121	3.01E-06
		26	Affx-87324563	2403259	0.221	0.662	0.145	5.06E-06
		12	Affx-93396222	10860972	0.349	-0.538	0.120	7.09E-06
		26	Affx-86915404	2971226	0.450	0.498	0.115	1.52E-05
		21	Affx-86977045	32739741	0.192	-0.626	0.146	1.69E-05
		25	Affx-86916185	14361093	0.181	0.628	0.147	1.81E-05
		26	Affx-87723009	2274470	0.285	0.582	0.136	1.85E-05
		26	Affx-87775881	2569233	0.267	-0.552	0.129	1.91E-05
		3	Affx-87597243	71065500	0.498	-0.496	0.116	2.01E-05
Shuffling	Male	7	Affx-87702070	30082074	0.177	0.253	0.053	2.08E-06
		15	Affx-87438278	97920722	0.201	0.208	0.049	1.94E-05
		10	Affx-87814965	96995476	0.399	0.180	0.042	1.94E-05
		10	Affx-87169893	17900600	0.567	-0.179	0.042	2.34E-05
		9	Affx-87137995	63788953	0.370	0.197	0.047	2.37E-05
		15	Affx-87191200	95892936	0.239	0.194	0.047	3.65E-05
		15	Affx-87285851	101670539	0.192	0.215	0.052	3.70E-05
		15	Affx-87285853	101671231	0.192	0.215	0.052	3.70E-05
		28	Affx-87026689	35657626	0.198	0.218	0.053	4.24E-05
		15	Affx-87320647	97937595	0.315	0.176	0.043	4.27E-05
Shuffling	Female	29	Affx-87275877	17198796	0.200	0.252	0.049	2.32E-07
		9	Affx-87805131	11090861	0.539	-0.198	0.040	5.58E-07
		7	Affx-87197793	30511902	0.196	-0.256	0.051	6.68E-07
		24	Affx-87658020	15513356	0.339	-0.182	0.040	4.46E-06
		14	Affx-87347934	62926154	0.440	-0.175	0.039	5.71E-06
		11	Affx-87566793	14623343	0.219	0.209	0.046	6.33E-06
		5	Affx-87225703	10195400	0.290	0.193	0.043	7.59E-06
		12	Affx-87008423	14255690	0.218	0.214	0.048	8.24E-06
		11	Affx-86997783	9768960	0.491	0.178	0.040	8.65E-06
		14	Affx-87808468	33998757	0.132	-0.242	0.055	9.73E-06

bp is the basepair position of the SNP marker, **Freq** is the allele frequency of the effect allele, **b** is the the effect estimate and **SE** the subsequent standard error of the effect estimate, **p** is the p value.

Table S3. Linkage mapping family structures.

**Family info**	**N**
Mean nr of offspring per family	100.5
Median nr of offspring per family	76
All four grandparents genotyped	4459
Range in nr of offspring per family	1-537
Total nr of families	5568
